# Locoregional Surgery for Infected Penile Squamous Cell Carcinoma With Bulky Nodal Disease: A Case Report

**DOI:** 10.7759/cureus.116132

**Published:** 2026-09-12

**Authors:** Tarcísio Gallucci, Fernando K Lerner, João Pedro T Mascarenhas, Alexandre Ferrari, João G Brunca, Felipe Sandoval, Roni C Fernandes

**Affiliations:** 1 Department of Surgery, Irmandade da Santa Casa de Misericórdia de São Paulo, São Paulo, BRA; 2 Department of Urology, Irmandade da Santa Casa de Misericórdia de São Paulo, São Paulo, BRA

**Keywords:** cn3 disease, locoregional surgery, penile cancer, sepsis, squamous cell carcinoma, tumor-associated infection, tumor necrosis, vascular complications

## Abstract

For chemotherapy-fit patients with clinical N3 (cN3) penile squamous cell carcinoma (SCC), contemporary guidelines favor neoadjuvant cisplatin- and taxane-based chemotherapy followed by consolidative surgery when feasible. Severe tumor-associated infection and necrosis may, however, temporarily alter this sequence.

A 53-year-old man with poorly differentiated penile SCC had previously undergone glansectomy and left inguinal lymphadenectomy, followed by one cycle of paclitaxel, ifosfamide, and cisplatin (TIP). After relocating to São Paulo, he was lost to oncologic follow-up and did not complete systemic treatment. On hospital day (HD) 1 of the current admission, he presented with extensive ulcerated and necrotic locoregional disease, purulent drainage, and sepsis. Initial management included meropenem, clinical stabilization, cross-sectional restaging, and multidisciplinary assessment while resumption of systemic therapy was considered. Imaging demonstrated bulky bilateral inguinal and pelvic nodal disease without radiologically evident distant metastases, consistent with cN3M0 disease. Persistent purulent drainage despite medical treatment led to operative management on HD26. The procedure combined resection of contiguous infected and malignant locoregional disease with an oncologic pelvic nodal dissection during the same anesthetic.

The postoperative course was complicated by wound dehiscence and arterial hemorrhage on postoperative day (POD) 16, requiring left external iliac artery angioplasty using polytetrafluoroethylene (PTFE). This was followed by *Pseudomonas aeruginosa* bacteremia with a highly restricted susceptibility profile, recurrent systemic inflammatory signs and wound drainage, PTFE removal with arterial ligation, progressive lower-limb ischemia, and death on POD32.

This case illustrates an uncommon scenario in which persistent tumor-associated infection altered the usual sequence of multimodal treatment and prompted combined infectious and oncologic locoregional surgery, followed by severe vascular and infectious morbidity.

## Introduction

Penile squamous cell carcinoma (SCC) is a rare malignancy in which lymph node involvement is a major determinant of prognosis [1]. For chemotherapy-fit patients with clinical N3 (cN3) disease, contemporary guidelines favor cisplatin- and taxane-based neoadjuvant chemotherapy followed by consolidative surgery in responders or patients without progression when resection is feasible [2-4]. Multidisciplinary care in experienced centers is particularly important in advanced penile cancer because optimal management may require coordinated systemic, surgical, and reconstructive strategies [5]. Paclitaxel, ifosfamide, and cisplatin (TIP) is among the best-studied regimens in this setting [2,6].

Extensive ulceration, necrosis, and tumor-associated infection may occasionally make immediate continuation of systemic treatment impracticable. We describe a patient with advanced cN3 penile SCC after interrupted systemic therapy who presented with sepsis and a persistent infected tumor burden and ultimately underwent complex locoregional surgery after initial medical management and multidisciplinary planning.

## Case presentation

Initial diagnosis and treatment

Approximately five months before the current admission, a 53-year-old man underwent glansectomy and left inguinal lymphadenectomy in Amapá, Brazil. Histopathological examination demonstrated poorly differentiated, infiltrative, and ulcerated SCC with approximately 40% tumor necrosis, urethral and corpus spongiosum invasion, perineural invasion, lymphovascular invasion, positive surgical margins, and metastases in all three examined lymph nodes. The outside pathology report classified the tumor as pT3pN2. Staging terminology in this report follows the AJCC/UICC eighth edition [7]. The descriptive pathology available from the initial specimen documented urethral and corpus spongiosum invasion but did not document corpus cavernosum invasion, limiting independent confirmation of the reported pT3 category.

Approximately four months before the current admission, TIP chemotherapy was initiated. The patient received one cycle before relocating to São Paulo, where he was lost to oncologic follow-up and did not complete the planned systemic treatment.

Admission and initial management

On hospital day (HD) 1, the patient was admitted to a quaternary referral center in São Paulo with severe local pain, weakness, and profuse purulent discharge from the genital and left inguinal regions.

At admission, his temperature was 38.6°C, heart rate was 115 beats/min, and blood pressure was 105/57 mmHg. Laboratory results at admission are summarized in Table 1. His Eastern Cooperative Oncology Group (ECOG) performance status was 1 [8]. His Sequential Organ Failure Assessment (SOFA) score was 3 [9], and sepsis was diagnosed according to the Third International Consensus Definitions for Sepsis and Septic Shock (Sepsis-3) [10]. Initial blood cultures were negative, and meropenem was initiated.

**Table 1:** Laboratory results at admission. Reference values correspond to the institutional laboratory reference ranges.

Laboratory parameter	Patient value	Reference value
Leukocyte count	17,500 cells/mm³	4,000-10,000 cells/mm³
C-reactive protein	26.5 mg/dL	<1.0 mg/dL
Serum creatinine	0.4 mg/dL	0.66-1.25 mg/dL

**Figure 1:**
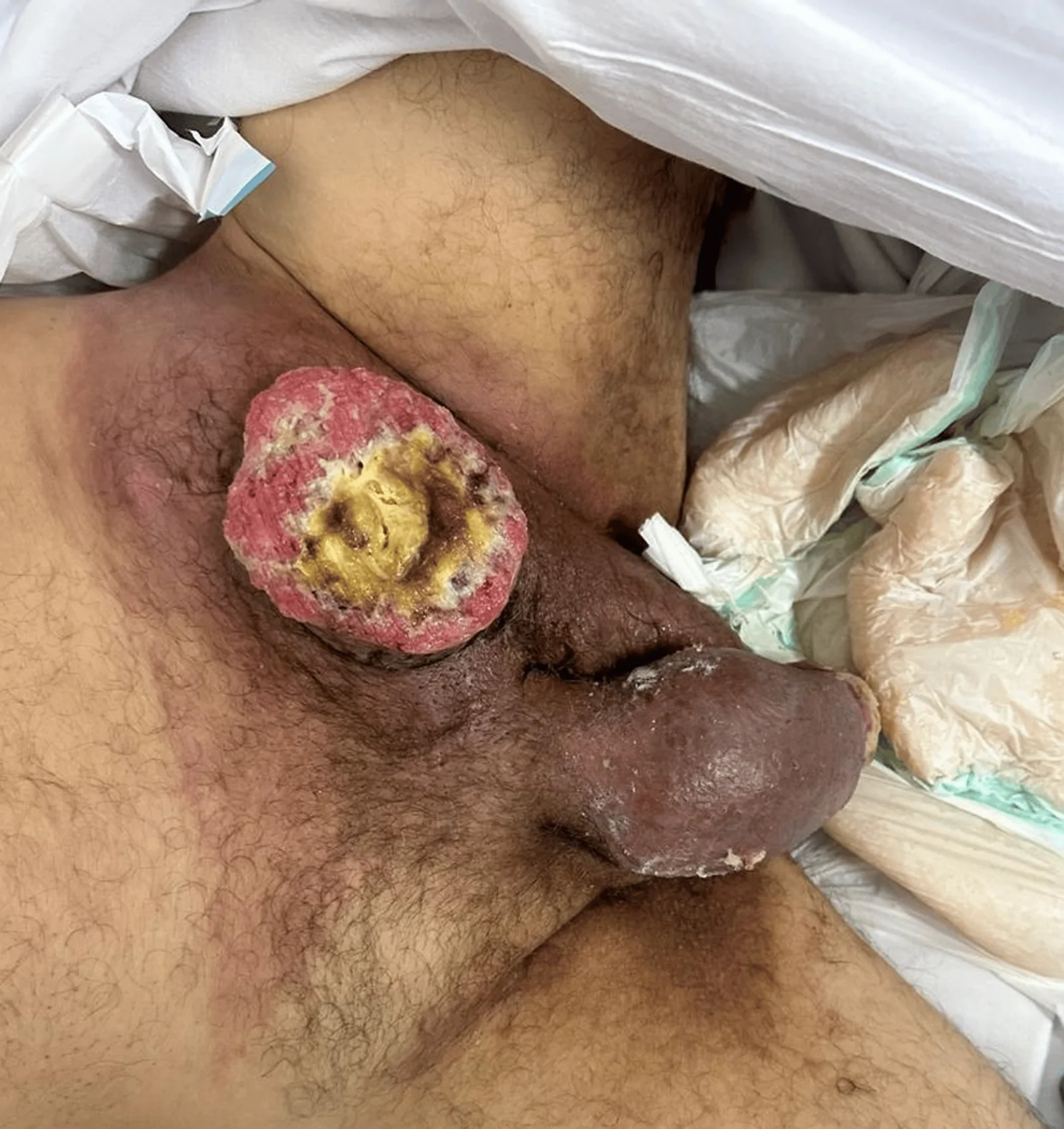
Clinical presentation at admission. Extensive ulcerated and exophytic left inguinal mass with central necrotic and fibrinous areas.

Physical examination demonstrated marked penile edema and a large exophytic and ulcerated left inguinal tumor with necrotic areas and abundant exudate (Figure 1). The residual penile stump was almost entirely covered by thick fibrinous-necrotic tissue.

Restaging and multidisciplinary treatment planning

Following admission, the patient underwent clinical stabilization and cross-sectional restaging while the feasibility of resuming systemic treatment was assessed.

**Figure 2:**
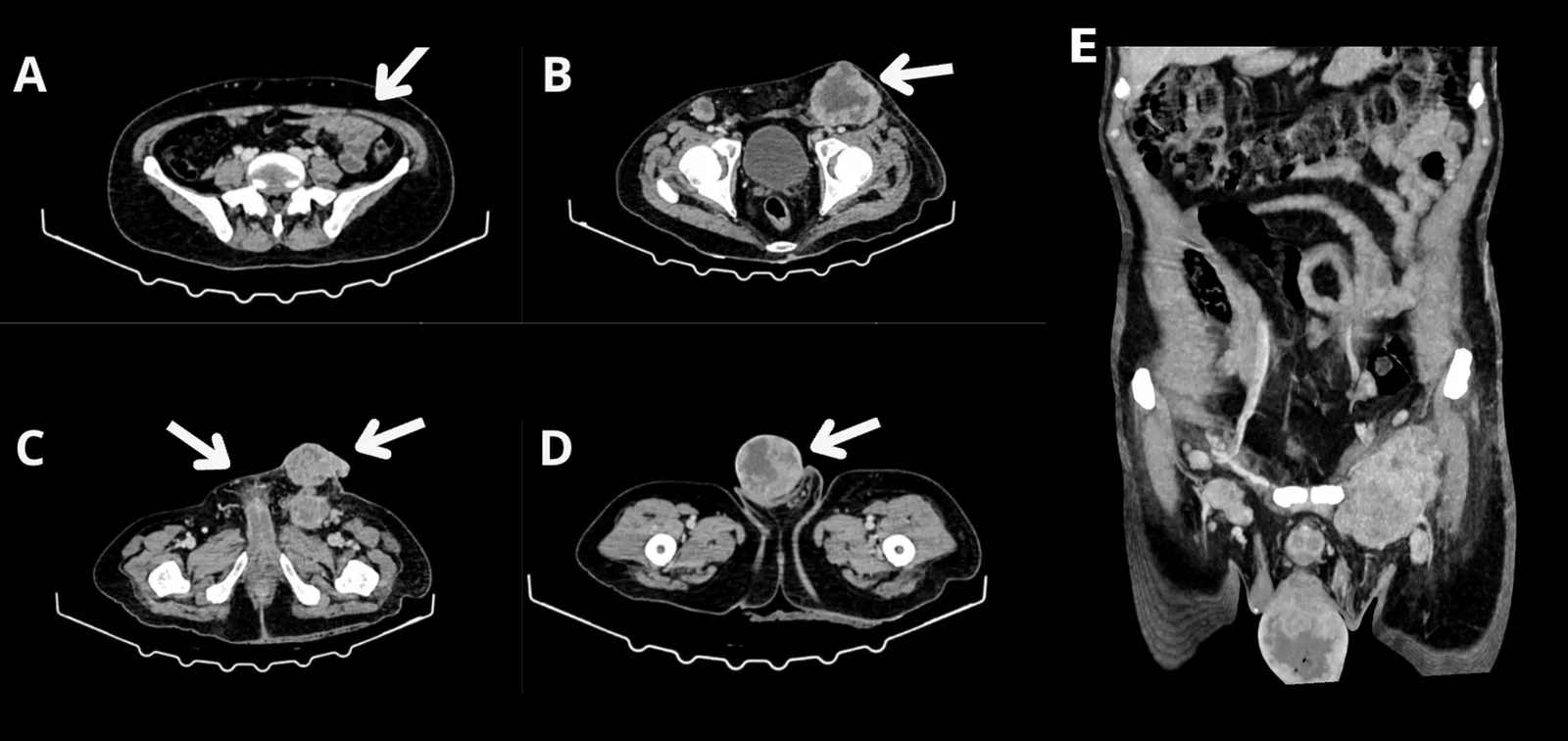
CT findings at restaging. (A) Lymphadenopathy at the left iliac bifurcation. (B) Bulky left-sided nodal disease. (C) Left inguinal nodal disease associated with penile involvement. (D) Penile tumoral infiltration. (E) Bulky left inguinal tumor mass with pelvic extension, also involving the penis and infiltrating the scrotal region. Arrows indicate the main sites of disease involvement. CT: Computed tomography.

Computed tomography of the chest, abdomen, and pelvis demonstrated extensive bilateral inguinal and pelvic nodal disease, including lymphadenopathy at the left iliac bifurcation, bulky left inguinal nodal disease, and penile and scrotal involvement (Figure 2). No radiologically evident distant metastases were identified, consistent with cN3M0 disease.

CT angiography was subsequently performed to better assess the relationship between the bulky inguinal disease and adjacent vascular structures as well as to assist operative planning.

Despite antimicrobial therapy and clinical stabilization, purulent drainage from the ulcerated and necrotic tumor persisted. The case was discussed by a multidisciplinary team involving urologic, plastic, and vascular surgeons. Resumption of systemic therapy was deferred because of the persistent infected and necrotic tumor burden. Radiotherapy was also discussed but was not considered an appropriate immediate strategy in the setting of active infection, an extensive exposed tumor bed, and marked tissue friability.

The operation was planned to combine management of the persistent infected tumor burden with locoregional oncologic resection. Contiguity between infected and malignant tissues made a limited local debridement unlikely to separate the two components. The bilateral pelvic lymphadenectomy was an oncologic component based on radiologically evident pelvic nodal disease and exceeded what was required for control of the local infection; it was performed during the same anesthetic. The team also considered that another major anesthetic exposure might be poorly tolerated in a patient who had recently presented with sepsis.

Locoregional surgery and final histopathology

On HD26, the patient underwent partial penectomy with coverage of the residual penile stump using scrotal tissue, left orchiectomy because of tumor involvement of the spermatic cord, transposition of the right testis to the inguinal region, bilateral obturator and external iliac lymphadenectomy, bilateral superficial and deep inguinal lymph node excision, and resection of the ulcerated left inguinal mass. Reconstruction included a left sartorius flap and a tensor fascia lata flap, with split-thickness skin grafting of the tensor fascia lata donor site.

The final histopathological examination demonstrated usual-type, poorly differentiated invasive SCC, histologic grade 3, with extensive tumor necrosis. The tumor involved the skin, corpora cavernosa, corpus spongiosum, urethra, and scrotal tissues. Lymphovascular and perineural invasion were present, and surgical margins were negative. p16 status was unavailable.

The initial outside pathology report, based on the earlier glansectomy and left inguinal lymphadenectomy specimen, had assigned pT3pN2. In contrast, the later surgical specimen obtained after progression of locoregional disease was reported as pT4N3M1. According to the AJCC/UICC eighth edition, scrotal extension and pelvic lymph node metastasis supported pT4 and pN3 disease, respectively [7]. However, neither the descriptive findings available from the final pathology report nor the preoperative imaging identified a distant metastatic site supporting pM1. Therefore, the reported M category could not be independently verified.

Had postoperative recovery permitted, further oncologic management would have been reassessed jointly with the medical oncology team. No definitive postoperative systemic or radiation treatment plan had been established at that time.

Postoperative vascular and infectious complications

**Figure 3:**
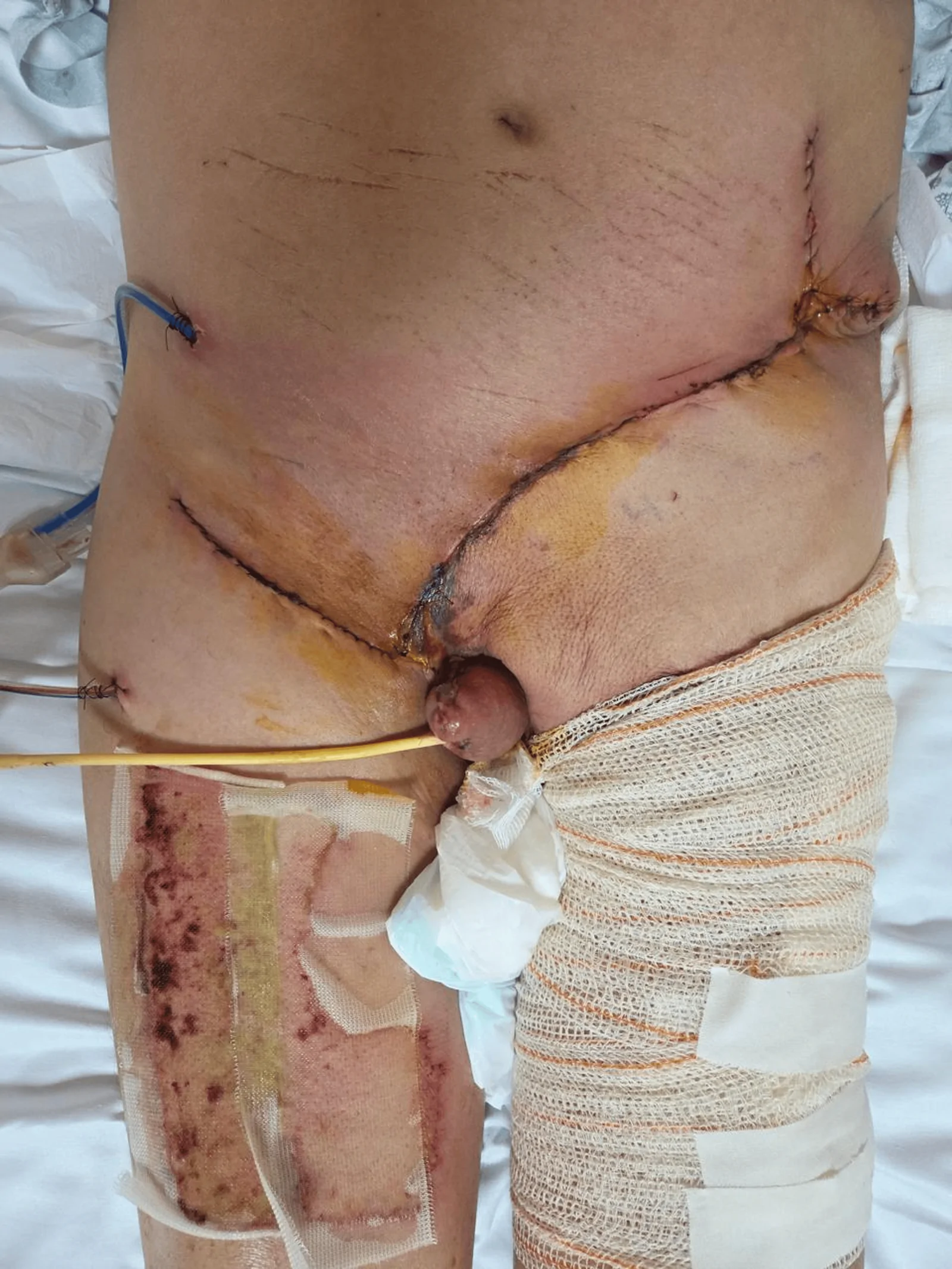
Early postoperative appearance after locoregional surgery. Clinical appearance on POD3 following extensive locoregional resection and reconstruction of the inguinal and genital operative fields.

The early postoperative appearance on POD3 is shown in Figure 3.

During the early postoperative period, dehiscence developed near the reconstructed penile stump, the left iliac crest, and the left inguinal surgical wound. The split-thickness skin graft also showed epidermolysis and marginal dehiscence. No clinical signs of infection were documented at that time. The patient remained on meropenem, with decreasing leukocyte count and C-reactive protein levels.

On POD16, the patient developed active arterial-appearing hemorrhage from the left inguinal surgical wound, associated with pallor, tachycardia, and borderline blood pressure. Direct compression and volume resuscitation were initiated, followed by rapid-sequence intubation and emergency operative management.

Angioplasty of the left external iliac artery was performed using polytetrafluoroethylene (PTFE) material documented as 6 mm, together with mechanical thromboembolectomy of the left lower extremity using a Fogarty catheter, mechanical debridement, and local wound management. Histopathological examination of a vascular specimen demonstrated recent and organizing thrombosis without reported neoplastic infiltration.

On POD17, one day after the vascular reconstruction, the patient developed fever, prompting collection of blood cultures. These subsequently grew *Pseudomonas aeruginosa*, with the isolate reported susceptible only to polymyxin B and amikacin. Meropenem was maintained, and the antimicrobial regimen was defined jointly with the infectious diseases team; polymyxin B was added.

Over the following days, fever, tachycardia, leukocytosis, and C-reactive protein elevation persisted, while the vascular surgical wound showed poor healing and ongoing drainage despite antimicrobial therapy. Subsequent control blood cultures were negative.

**Figure 4:**
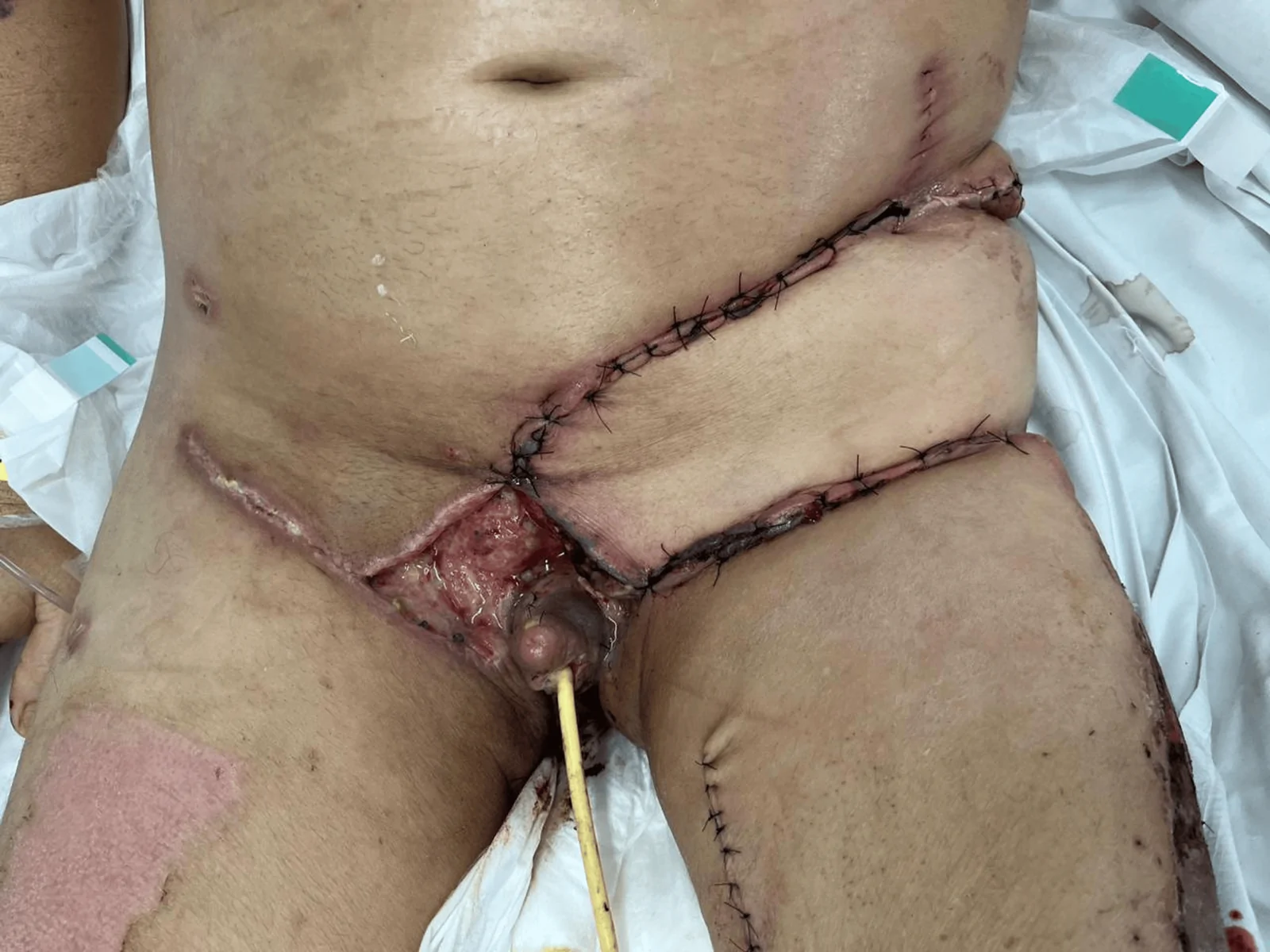
Wound appearance after vascular surgical intervention. Clinical appearance following vascular reintervention, demonstrating left inguinal wound dehiscence and impaired healing of the reconstructive site.

The wound appearance after vascular surgical intervention is shown in Figure 4.

By POD28, persistent systemic inflammatory signs and purulent drainage from the vascular surgical wound raised concern for infection involving the vascular reconstruction. The PTFE material was removed, and proximal and distal ligation of the left external iliac artery was performed together with local wound management and flap closure.

During anesthetic induction on POD28, the patient developed cardiac arrest, with return of spontaneous circulation after one cycle of cardiopulmonary resuscitation. He remained hemodynamically unstable during the procedure and required escalating vasopressor support.

Progressive clinical signs of left lower-limb ischemia subsequently developed. Transfemoral amputation was recommended on POD30 but was not performed following shared decision-making with the patient's family. The patient remained critically ill with progressive hemodynamic deterioration in the setting of severe postoperative vascular and infectious complications and died on POD32.

A summary of the patient's clinical course is provided in Table 2.

**Table 2:** Clinical timeline. HD: Hospital day; POD: Postoperative day; TIP: Paclitaxel, ifosfamide, and cisplatin; CT: Computed tomography; PTFE: Polytetrafluoroethylene.

Time point	Event
~5 months before HD1	Glansectomy and left inguinal lymphadenectomy
~4 months before HD1	One cycle of TIP chemotherapy
Pre-admission interval	Relocation to São Paulo and loss to oncologic follow-up
HD1	Admission with infected, necrotic locoregional disease and sepsis; meropenem initiated
HD1-HD25	Cross-sectional restaging, CT angiography, medical management, and multidisciplinary planning; purulent drainage persisted
HD26/POD0	Combined locoregional surgery, oncologic pelvic nodal dissection, and reconstruction
POD16	Arterial hemorrhage and left external iliac artery PTFE angioplasty
POD17	Fever prompted blood cultures; *Pseudomonas aeruginosa* isolated, susceptible only to polymyxin B and amikacin; meropenem maintained and polymyxin B added under infectious diseases guidance
~POD18 onward	Persistent systemic inflammatory signs, poor wound healing, and drainage despite antimicrobial therapy; subsequent control blood cultures were negative
POD28	PTFE removal and left external iliac artery ligation; cardiac arrest during anesthetic induction with return of spontaneous circulation
POD30	Transfemoral amputation recommended but not performed
POD32	Death

## Discussion

For chemotherapy-fit patients with cN3 penile SCC, contemporary guidelines favor neoadjuvant cisplatin- and taxane-based chemotherapy followed by consolidative surgery in responders or patients without progression when resection is feasible [2-4]. TIP remains one of the best-studied regimens in this setting [2,6]. Routine surgery-first management of cN3 disease is generally discouraged, and radical inguinal and pelvic lymphadenectomy can carry substantial wound and systemic morbidity [2,11]. In this patient, ECOG performance status was 1 [8] and serum creatinine was 0.4 mg/dL at admission; systemic therapy was deferred because of active sepsis and the persistent infected tumor burden rather than a documented baseline performance status or renal limitation.

The distinguishing feature of this case was persistent tumor-associated local infection despite antimicrobial treatment and initial clinical stabilization. The operation was not limited to infection control: resection of contiguous infected and malignant tissues was combined with bilateral pelvic lymphadenectomy performed for oncologic intent because pelvic nodal disease was evident on imaging. The pelvic dissection therefore exceeded what was necessary to treat the local infection. The decision to complete both components during the same anesthetic also reflected concern about the patient's ability to tolerate another major operative exposure after presenting with sepsis. Had postoperative recovery permitted, subsequent systemic or radiation treatment would have been reassessed jointly with medical oncology rather than being predetermined. This individualized sequence should not be interpreted as support for routine upfront surgery in cN3 disease.

The early postoperative course was marked by dehiscence involving the reconstructed penile, left iliac, and left inguinal operative sites and by skin-graft epidermolysis, without documented clinical infection, while leukocyte count and C-reactive protein levels were decreasing. The initial groin reconstruction had included a left sartorius flap. Arterial hemorrhage nevertheless occurred on POD16. The mechanism of this event cannot be established from the available records. Histopathological examination of the vascular specimen demonstrated thrombosis without neoplastic infiltration, and the relative contributions of surgical manipulation, inflammation, tissue friability, thrombosis, and the local oncologic field cannot be distinguished.

After vascular reconstruction with PTFE, fever on POD17 prompted blood cultures that grew *P. aeruginosa* susceptible only to polymyxin B and amikacin. Meropenem was maintained, polymyxin B was added under infectious diseases guidance, and subsequent control blood cultures were negative. Systemic inflammatory signs, poor wound healing, and drainage nevertheless persisted. The subsequent removal of the prosthetic material, local wound management, and arterial ligation represented a definitive operative approach to suspected extracavity vascular graft infection. Contemporary European Society for Vascular Surgery guidance supports multidisciplinary management, culture-directed antimicrobial therapy, and individualized operative treatment of extracavity vascular graft and endograft infection, including removal of infected prosthetic material when feasible [12]. Muscle-flap coverage is an established reconstructive option for deep groin infection following vascular surgery, although the supporting evidence is limited [13].

This case illustrates the difficulty of balancing oncologic treatment, management of persistent local infection, reconstructive requirements, and perioperative risk in advanced penile cancer. It also underscores the importance of continuity of oncologic care, early multidisciplinary referral, and management in experienced centers before ulceration, infection, and bulky nodal disease substantially narrow the available therapeutic options [5].

Limitations

This report is limited by its single-patient, retrospective nature and its reliance on available medical-record documentation. No deep-tissue culture from the initial infected tumor was obtained, p16 status was unavailable, and discrepancies in external and final pathological staging limited independent reconciliation of all TNM categories. The exact mechanism of the arterial hemorrhage could not be established. The detailed vascular operative report was unavailable, preventing confirmation of the precise PTFE configuration and operative rationale. These limitations preclude causal attribution of the vascular and infectious complications to any single factor.

## Conclusions

This case illustrates an uncommon clinical situation in which persistent tumor-associated infection prevented immediate continuation of systemic treatment despite antimicrobial therapy and clinical stabilization. After staging and multidisciplinary evaluation, surgery combined management of contiguous infected and malignant locoregional disease with an oncologic pelvic nodal dissection during the same anesthetic.

The subsequent wound, vascular, and infectious complications emphasize the substantial morbidity of extensive surgery in this setting. Management should remain individualized and multidisciplinary, with contemporary multimodal therapy remaining the preferred framework for appropriate patients with cN3 penile cancer.
